# Age estimation using dental and hand-wrist radiography among a sample of Egyptian children

**DOI:** 10.1038/s41598-024-83638-3

**Published:** 2025-01-07

**Authors:** Rania Abd Elmaguid Kaka, Marwa Ismail Mohamed Haiba, Abeer Abd Elmoneim Sheta, Naglaa Hassan Salama, Nagwa Mostafa Enany

**Affiliations:** 1 Forensic Medicine and Clinical Toxicology Department, Faculty of Medicine, Alexandria University, Alexandria, Egypt; 2 Orthodontics, Faculty of Dentistry, Alexandria University, Alexandria, Egypt

**Keywords:** Willems method, Dental age, Cameriere’s method, Greulich and Pyle atlas, Identification

## Abstract

The current study aimed to evaluate the accuracy of Willems, Cameriere’s and Greulich and Pyle method in age estimation among a sample of Egyptian children aged 8–16 years based on analysis of 140 panoramic dental X-ray and hand-wrist radiographs (70 girls and 70 boys). Using Willems method, the mean dental age underestimated chronological age by (0.20 ± 0.91 years) for boys and by (0.24 ± 1.33 year) for girls; these differences were not statistically significant. Also, Cameriere’s method underestimated chronological age in both sexes by mean difference of (1.10 ± 1.22 year) in boys and (1.13 ± 1.31 year) in girls; these differences were statistically significant. Regarding Greulich and Pyle atlas, the mean of skeletal age was overestimated when compared to the real age in boys with a mean difference (0.04 ± 0.86 year). In girls; the age was underestimated by a mean difference of (0.15 ± 1.32 years) when compared to chronological age; these differences were not statistically significant. Comparing the two dental methods, the mean absolute error using Willems method was less than one year (0.94 and 0.96 years) in boys and girls respectively, while in Cameriere’s method, it was more than one year in both groups. Moreover; the mean absolute error using Greulich and Pyle atlas was less than two years (1.02 and 1.38 years) in boys and girls respectively. Since Willems and the atlas methods were more accurate, the combination of both of them could be used for age estimation in the Egyptian population.

**Supplementary Information:**

The online version contains supplementary material available at 10.1038/s41598-024-83638-3.

## Introduction

Age estimation is a critical task in forensic examination which needs precise methodologies. The lack of a single approach that could be used across all ages and populations has prompted ongoing studies in this area. Since different nations have distinct maturation and developmental patterns, it would seem reasonable to assume that there is no uniform approach for estimating age that could be applied to all populations^1,2^.

Numerous hypotheses demonstrated differences in development across different populations because of the interaction of genetic and environmental variables. Moreover, many populations’ demographic characteristics, as well as their physical profiles, have changed as a result of increased globalization. Various parameters are changing as well, and they may be linked to changes in dietary status, socioeconomic level, and genetic mixing^3^.

For adults, a wide range of criminal, legal, and civil concerns depend on accurate age estimation. In minors, cases of adoption, criminal allegations, child labor, and child pornography need exact age determination as well^2,3^. According to the United Nations; At least 89.3 million people around the world have been forced to flee their homes as a result of conflict and persecution at the end of 2021, about 36.5 million of whom are children below 18 years old^4^.

In Egypt, despite the absence of clear data on street children, several socio-economic indices imply that the situation is expanding, particularly in large cities. Considering this development, age determination in criminal proceedings has become of an increasing necessity in forensic practice. The age of relevance in criminal proceedings in Egypt is set at seven to twenty-one years old^5^. Therefore, it is critical to determine age using precise and reliable procedures to determine whether juvenile or adult penal systems will be applied^6^.

The predicted dental age based on dental mineralization/development is fairly accurate as it is less affected by external factors compared to teeth eruption process. Another reason why tooth mineralization is commonly preferred is that; it is a continuous phenomenon that can be detected each quarter of the year radiologically^3,4,6^.

In 1973, Demirjian et al. presented a method that estimated chronological age according to the stages of dental development of the seven left permanent mandibular teeth using data collected from French Canadian children. Each stage is given a score, and the aggregate of the values is used to calculate the subject’s dental maturity^7^. When this method was applied to different populations, it revealed an overestimation of dental age by up to a year. This could be attributed to differences in genetics, geography and climate, sample size, and methods of analysis^6^.

Several authors subsequently modified the original method to obtain more accurate results when applied to different populations. Therefore, it is advised that dental age should be determined for each population based on their own specific standards^6^.

In 2001, Willems et al.^8^ aimed to simplify the original version by reducing the conversion steps, and produced a modified version of Demirjian’s scoring system employing Belgian Caucasian population^8^. Willems approach was applied to various populations and produced accurate and trustworthy findings^9^.

Cameriere et al. chose a completely different approach using a sample from some European countries. The approach utilized digital panoramic radiographs as well. This method is based on the measurement of the open apices of the left seven permanent mandibular teeth^10^.

For sub-adult populations, age determination can be done using ossification centers fusion as it is the final event occurring within the skeleton development. Although various methods are proposed for age determination using radiographs of the carpal bones to assess skeletal maturity, the Greulich and Pyle method is outstanding, quick, and simple^11,12^. This method relies on a visual inspection, which compares the radiograph to a pattern described in an atlas based on a sample of North American children^13^.

This study aimed to evaluate the accuracy of applying Willems and Cameriere’s as well as Greulich and Pyle’s atlas in age estimation among Egyptian children and to identify the most reliable method for accurate age estimation. Additionally, to establish an anthropological method from literature utilizing different parameters rather than just one to yield more accurate results in Egyptians (< 18 years old).

## Subjects and methods

This prospective study was conducted on 140 orthopantomograms and hand wrist X-rays of 70 randomly selected boys and 70 randomly selected girls with age range (8–18 years) in the Department of Orthodontics in a tertiary health care hospital in Alexandria, Egypt. The study was conducted on patients attending Orthodontics clinics at Faculty of Dentistry, Alexandria University. The study sample was a good representative for children from most of Egypt areas, as this is a tertiary hospital that directly serves most of the Northern and Western Egyptian governorates and also it is the main referral hospital for many distant governments. Panoramic radiography was done for studied patients, as well as, hand-wrist X-ray as part of the investigation and orthodontic treatment protocol.

Samples were divided into ten groups according to chronological age (CA) (one group per year). However: the number of children is not equal in all age groups as this sample was randomly collected during a specific period of time (9 months) and included all children matching the inclusion criteria. The sex and age distributions of each group are presented in Table 1.

**Table 1:** Distribution of Studied Individuals by Age and Sex (n = 140)^14^.

Age group	Age range	Sex	Number%	Mean age ± SD (years)	Median (years)
8	8–8.9	Males	13(18.57%)	8.58 ± 0.29	8.60
		Females	11(15.72%)	8.42 ± 0.34	8.30
9	9–9.9	Males	10(14.28%)	9.33 ± 0.26	9.30
		Females	6(8.57%)	9.48 ± 0.31	9.55
10	10–10.9	Males	4(5.72%)	10.12 ± 0.05	10.1
		Females	4(5.72%)	10.15 ± 0.17	10.15
11	11–11.9	Males	8(11.43%)	11.47 ± 0.26	11.5
		Females	9(12.85%)	11.36 ± 0.2	11.4
12	12–12.9	Males	6(8.57%)	12.60 ± 0.39	12.8
		Females	8(11.42%)	12.12 ± 0.12	12.1
13	13–13.9	Males	12(17.15%)	13.17 ± 0.19	13.15
		Females	4(5.71%)	13.2 ± 0.17	13.25
14	14–14.9	Males	3 (4.28%)	14.76 ± 0.15	14.8
		Females	12(17.15%)	14.36 ± 0.27	14.3
15	15–15.9	Males	6(8.57%)	15.13 ± 0.05	15.1
		Females	8(11.43%)	15.33 ± 0.31	15.25
16	16–16.9	Males	0	0	0
		Females	6(8.57%)	16.28 ± 0.31	16.1
17	17–17.9	Males	8(11.43%)	17.37 ± 0.39	17.2
		Females	2(2.86%)	17.00	17
Total	8–17.9	Males	70(100%)	12.07 ± 2.89	11.95
		Females	70(100%)	12.42 ± 2.72	12.10

## Selection criteria

Panoramic radiography as well as hand-wrist X-ray were done for all studied patients as part of the investigations and orthodontic treatment protocol.

*The inclusion criteria included*: (1) patients with documented date of birth and date of radiography (2) clear and high-quality radiographs, (3) healthy individuals with a complete set of seven left permanent mandibular teeth (erupted or unerupted) and with physical development appropriate to their age (using standard growth charts). (4) no past medical history or surgery that could affect the eruption or mineralization of the seven left permanent mandibular teeth.

*The exclusion criteria included:* (1) Orthopantomogram with missing mandibular permanent teeth on both sides of the mandible, except the third molar; (2) skeletal, facial, or dental congenital anomalies. (3) children with systemic disease; gross pathology; local trauma or tumor in the mandible.

*Ethical approval:* The study was conducted after Ethical clearance was obtained from the Ethical Committee of the affiliated institution. Written informed consent was obtained from each parent/ guardian of the studied children and they were informed that their children’s dental records and radiographs would be used only for research and the confidentiality of the records would be preserved. Authors confirm that all methods were performed in accordance with the relevant guidelines and regulations.

## Calculation of chronological age

Chronological age (CA) was considered as the gold standard and this was calculated in decimal years. The dates of birth and dates of radiographs were obtained. A function of Microsoft Excel was used to calculate the difference between the recorded date of birth and the date on which the panoramic or hand wrist radiograph was made, to obtain the CA in decimal years.

## Calculation of dental age (DA) and skeletal age

### The dental methods

#### Willems method^8^

Evaluation of the left 7 mandibular teeth for both boys and girls was done according to developmental (calcification) stages of Willems method. Dental age was measured directly by summation of the maturity scores of the 7 left mandibular teeth for males and females’ tables. Dental age was compared statistically to chronological age.

#### Cameriere’s method^10^

Dental age in Cameriere’s method was determined based on the relationship between age and the measurement of the open apices in teeth using the same left seven mandibular teeth as explained in the original study.

### Skeletal age (Greulich and Pyle atlas)^13^

Left hand-wrist radiograph was taken for each subject. The landmarks within the radiograph were closely examined and compared with the standard skeletal age plates of Greulich and Pyle atlas and bone age was assigned.

### Statistical analysis

All the analyses and calculations were performed using Statistical Package for Social Science (SPSS, version 26, IBM Corp., Chicago, Il, USA). The normality of the continuous variables was checked using the Kolmogorov–Smirnov test. Continuous variables are presented as means and standard deviations (SD). Categorical variables are displayed as frequencies and percentages. Wilcoxon signed-rank test was applied to compare means of chronological age and means of estimated dental and skeletal ages. A p-value ≤ 0.05 was considered statistically significant.

## Results

### Dental methods

#### Comparison of results obtained using Willems and Cameriere’s methods

Both methods showed underestimation of age when compared to the chronological age in both sexes. In boys the mean estimated dental age using Willems method was 11.87 ± 2.64 with mean difference (0.20 ± 0.91 year) compared to the chronological age. Willems method underestimated the age in all age groups except in age group (9, 11 and 13). As the sample was collected patients from different governorates and Egyptian country side, underestimation was not found in these age groups which could be attributed to different genetic or developmental background.

**Fig. 1:**
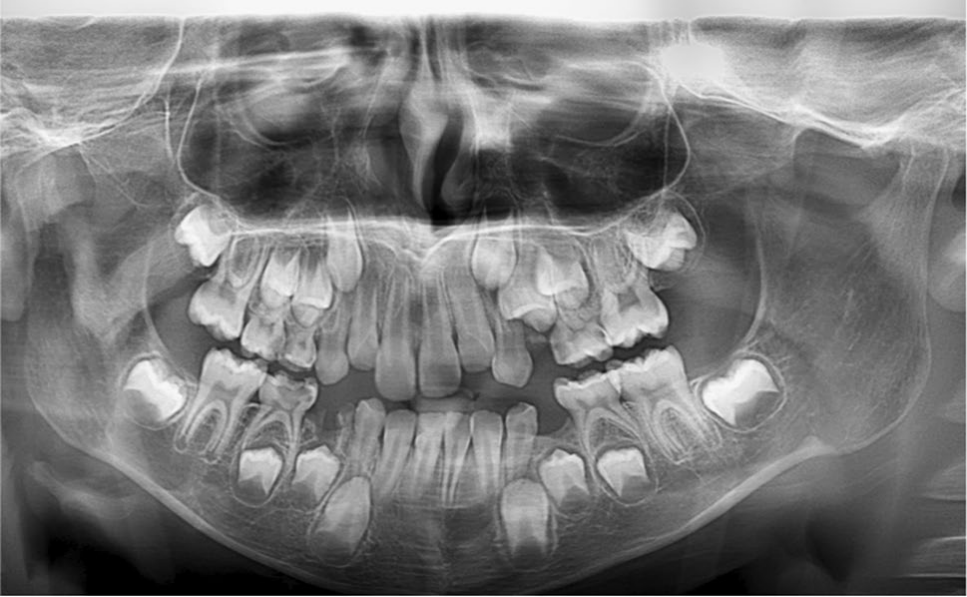
Panoramic image of male patient (Age 8 years and 6 months) Age according to Willem’s method is 8.02 years. Age according to Cameriere’s method is 7.05 years.

Wilcoxon signed rank test revealed statistically significant differences between means of chronological age and estimated dental age in the following age groups (8, 15, and 17) where P ≤ 0.05. The mean absolute error (MAE) was less than one year among the whole male sample (0.94 years) as shown in Table 2 and Fig. 1.

**Table 2:** Comparison between chronological age and dental age estimated by Willems method in males (n = 70).

Age group	Chronological age (years)		Estimated dental age (years)		Mean difference (years)	P value#	Mean absolute error (MAE)
	Mean ± SD	Median (years)	Mean ± SD	Median (years)			
8	8.58 ± 0.29	8.60	8.29 ± 0.37	8.30	− 0.29 ± 0.39	0.028*	0.41
9	9.33 ± 0.26	9.30	9.86 ± 0.70	9.6	+ 0.53 ± 0.83	0.059	0.76
10	10.12 ± 0.05	10.1	9.66 ± 0.66	9.67	− 0.46 ± 0.70	0.461	0.60
11	11.47 ± 0.26	11.5	11.60 ± 0.52	11.55	+ 0.13 ± 0.55	0.575	0.49
12	12.60 ± 0.39	12.8	12.42 ± 1.19	12.74	− 0.18 ± 0.83	0.463	0.65
13	13.17 ± 0.19	13.15	13.58 ± 2.11	13.68	+ 0.41 ± 2.02	0.239	1.55
14	14.76 ± 0.15	14.8	13.64 ± 0.87	14.15	− 1.12 ± 0.99	0.109	1.12
15	15.13 ± 0.05	15.1	13.51 ± 1.12	14.15	− 1.62 ± 1.14	0.028*	1.62
16	0	0	0	0	0	0	−
17	17.37 ± 0.39	17.2	16.02 ± 0.01	16.03	− 1.35 ± 0.39	0.012*	1.34
Total	12.07 ± 2.89	11.95	11.87 ± 2.64	11.95	− 0.20 ± 0.91	0.059	0.94

^#^: Wilcoxon Signed Rank test; *: Statistically significant p ≤ 0.05.

Headings in the table is written in (bold).

Regarding girls; Table 3 revealed that this method also underestimated age in females with mean difference (0.24 ± 1.33 year) compared to the chronological age. Underestimation of the age in all groups was observed except in group (9, 11, 12 and 13) where age overestimation was noticed. The difference between the calculated dental age and real age was significant only in age group 16 where P = 0.03. The MAE was also less than one year among the whole female sample (0.96 years).

**Table 3:** Comparison between chronological age and dental age estimated by Willems method in females (n = 70).

Age group	Chronological age (years)		Estimated dental age (years)		Mean difference (years)	P value#	Mean absolute error (MAE)
	Mean ± SD	Median (years)	Mean ± SD	Median (years)			
8	8.42 ± 0.34	8.3	8.31 ± 0.20	8.15	− 0.11 ± 0.50	0.269	0.40
9	9.48 ± 0.31	9.55	9.68 ± 0.63	10.03	+ 0.20 ± 0.37	0.248	0.35
10	10.15 ± 0.17	10.15	9.98 ± 0.55	9.64	− 0.17 ± 0.38	0.093	0.62
11	11.36 ± 0.2	11.4	11.71 ± 1.79	12.6	+ 0.35 ± 1.69	0.406	1.42
12	12.12 ± 0.12	12.1	12.29 ± 2.43	11.83	+ 0.17 ± 2.50	1.000	2.02
13	13.22 ± 0.17	13.25	15.15 ± 0.01	15.79	+ 1.93 ± 0.17	0.168	2.56
14	14.36 ± 0.27	14.3	13.52 ± 1.82	12.89	− 0.84 ± 1.85	0.099	1.85
15	15.33 ± 0.31	15.25	14.57 ± 1.34	14.81	− 0.76 ± 1.12	0.123	0.98
16	16.28 ± 0.31	16.1	15.79 ± 0.00	15.79	− 0.49 ± 0.37	0.027*	0.49
17	17.00 ± 0.00	17	15.79 ± 0.00	15.79	− 1.21 ± 0.00	0.157	1.21
Total	12.42 ± 2.72	12.10	12.18 ± 2.88	11.76	− 0.24 ± 1.33	0.239	0.96

Headings in the table is written in (bold).

^#^: Wilcoxon Signed Rank test; *: Statistically significant p ≤ 0.05.

Cameriere method also underestimated the age in both sexes by mean difference of (1.10 ± 1.22 year) in boys and (1.13 ± 1.31 year) in girls (Tables 4 and 5). Underestimation of age in boys was noticed in all age groups except in age group 10 where overestimation was noticed. A significant difference was noted between calculated dental age and chronological age in the following age groups 8,11,12,15, 17.

**Table 4:** Comparison between chronological age and dental age estimated by Cameriere method in males (n = 70).

Age group	Chronological age (years)		Estimated dental age (years)		Mean difference (years)	P value #	Mean absolute error (MAE)
	Mean ± SD	Median (years)	Mean ± SD	Median (years)			
8	8.58 ± 0.29	8.60	7.78 ± 0.38	7.90	− 0.80 ± 0.45	0.002*	0.80
9	9.33 ± 0.26	9.30	9.05 ± 0.74	8.92	− 0.28 ± 0.87	0.168	0.68
10	10.12 ± 0.05	10.1	10.36 ± 0.16	10.36	+ 0.24 ± 0.14	0.066	0.23
11	11.47 ± 0.26	11.5	10.70 ± 0.42	10.79	− 0.77 ± 0.44	0.012*	0.77
12	12.60 ± 0.39	12.8	11.50 ± 0.98	11.76	− 1.10 ± 0.63	0.027*	1.09
13	13.17 ± 0.19	13.15	12.42 ± 1.23	12.51	− 0.75 ± 1.15	0.158	1.05
14	14.76 ± 0.15	14.8	13.55 ± 0.90	14.06	− 1.21 ± 1.03	0.109	1.21
15	15.13 ± 0.05	15.1	12.85 ± 1.43	13.46	− 2.28 ± 1.45	0.028*	2.28
16	0	0	0	0	0	0	−
17	17.37 ± 0.39	17.2	14.18 ± 0.35	14.06	− 3.19 ± 0.62	0.012*	3.18
Total	12.07 ± 2.89	11.95	10.97 ± 2.30	10.81	− 1.10 ± 1.22	< 0.001*	1.23

Headings in the table is written in (bold).

^#^: Wilcoxon Signed Rank test; *: Statistically significant p ≤ 0.05.

**Table 5:** Comparison between chronological age and dental age estimated by Cameriere method in females (n = 70).

Age group	Chronological age (years)		Estimated skeletal age (years)		Mean difference (years)	P value#	Mean absolute error (MAE)
	Mean ± SD	Median (years)	Mean ± SD	Median (years)			
8	8.58 ± 0.29	8.60	8.62 ± 0.1.19	8.00	+ 0.04 ± 1.18	0.807	0.87
9	9.33 ± 0.26	9.30	10.50 ± 1.35	10.00	+ 1.17 ± 1.46	0.008*	1.17
10	10.12 ± 0.05	10.1	10.50 ± 0.57	10.5	+ 0.38 ± 0.55	0.461	0.47
11	11.47 ± 0.26	11.5	12.38 ± 1.40	13	+ 0.91 ± 1.36	0.161	1.47
12	12.60 ± 0.39	12.8	12.67 ± 0.51	13	+ 0.07 ± 0.79	0.596	0.63
13	13.17 ± 0.19	13.15	13.17 ± 0.38	13	+ 0.00 ± 0.43	0.673	0.27
14	14.76 ± 0.15	14.8	16.67 ± 2.30	18	+ 1.9 ± 2.43	0.285	2.50
15	15.13 ± 0.05	15.1	13.00 ± 0.89	13	− 2.13 ± 0.87	0.027*	2.13
16	0	0	0	0	0	0	−
17	17.37 ± 0.39	17.2	16.75 ± 1.16	17	− 0.62 ± 1.10	0.204	0.90
Total	12.07 ± 2.89	11.95	12.11 ± 2.73	13.0	+ 0.04 ± 0.86	0.354	1.02

Headings in the table is written in (bold).

^#^: Wilcoxon Signed Rank test; *: Statistically significant p ≤ 0.05.

This method underestimated the age in girls in all age groups except group 13. A significant difference was found between the calculated dental age and the chronological age in group 8, 14, 15, and 16 where P < 0.05.

Regarding Cameriere method, the MAE was more than one year among the whole male sample (1.23 years) and among the whole female sample (1.40) years.

Regarding girls; Table 3 revealed that this method also underestimated age in females with mean difference (0.24 ± 1.33 year) compared to the chronological age. Underestimation of the age in all groups was observed except in group (9, 11, 12 and 13) where age overestimation was noticed. The MAE was also less than one year among the whole female sample (0.96 years).

Cameriere method also underestimated the age in both sexes by a mean difference of (1.10 ± 1.22 years) in boys and (1.13 ± 1.31 years) in girls (Tables 4 and 5). Underestimation of age in boys was noticed in all age groups except in age group 10 where overestimation was noticed. This method underestimated the age in girls in all age groups except group 13. Regarding the Cameriere method, the MAE was more than one year among the male sample (1.23 years) as well as the female sample (1.40 years).

Comparing MAE between the two dental methods showed that Willims method is more accurate in both boys and girls as the MAE is less than one year in both. On the other hand, Cameriere method was considered less accurate as the MAE was more than one year in both boys and girls.

## Skeletal method (bone age)

**Fig. 2:**
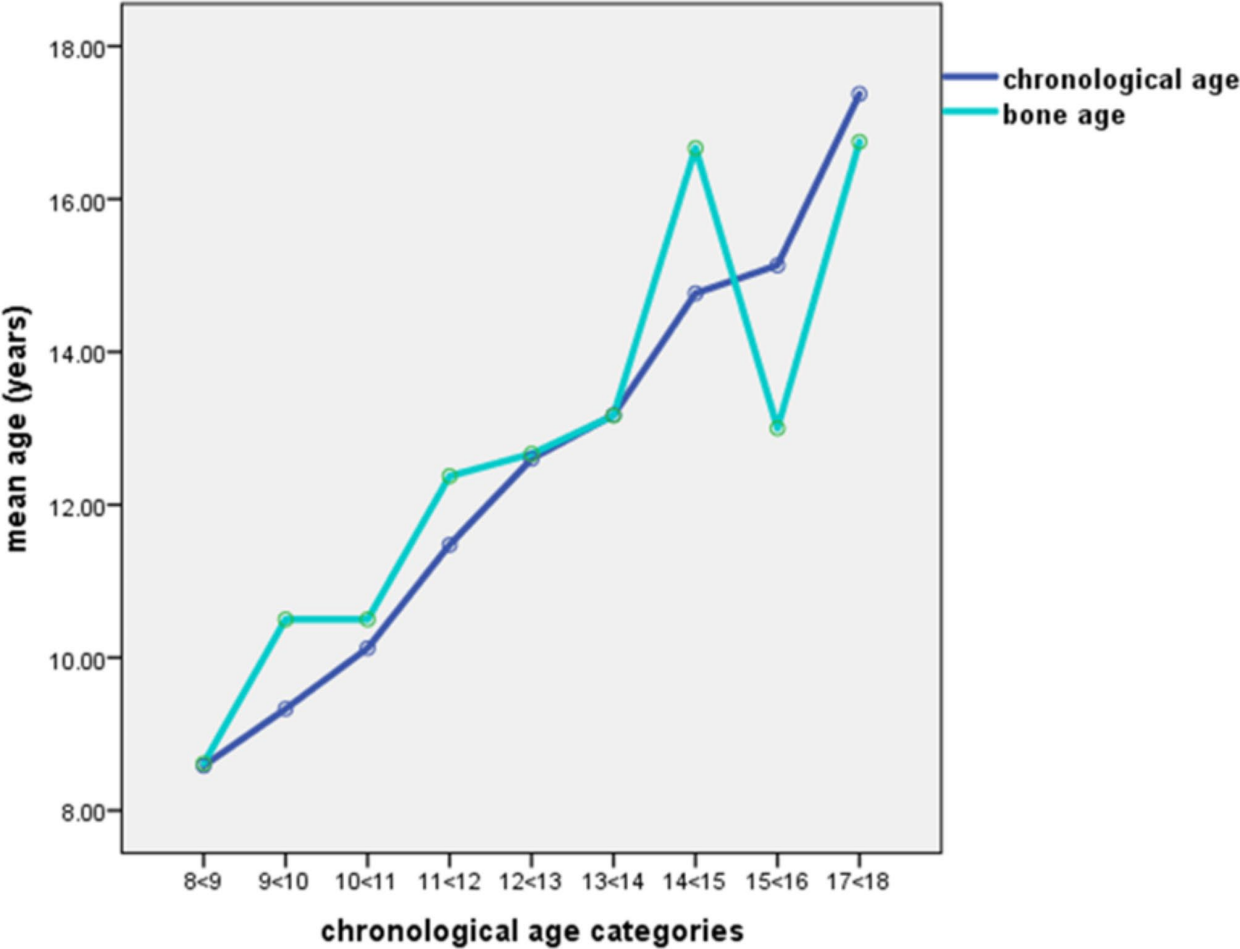
Comparison between chronological age and skeletal age estimated by Greulich and Pyle method among males.

**Fig. 3:**
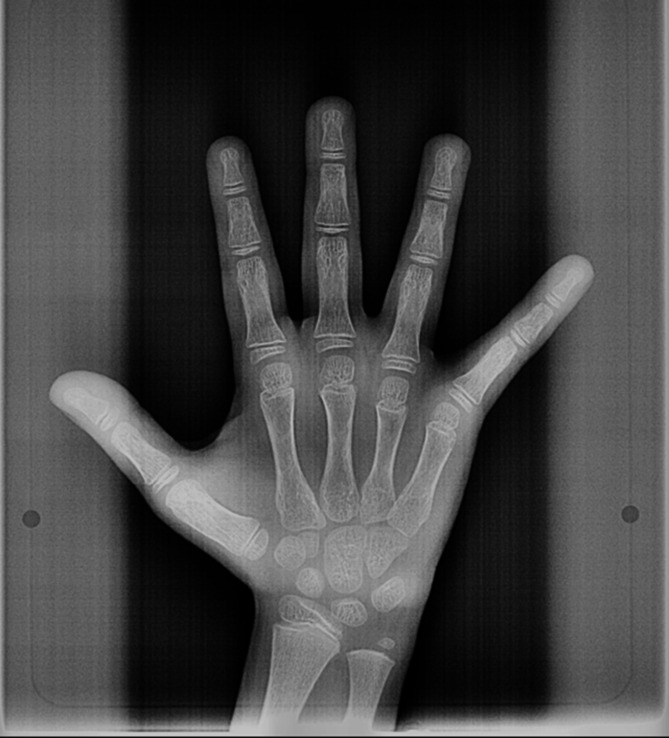
Hand wrist X- ray of male patient (Age 8 years and 6 months) According to standard plates of Greulich and Pyle atlas age could be 10 years.

Figures 2 and 3 demonstrated the comparison between chronological age and skeletal age (Greulich and Pyle) in males. The mean of skeletal age was overestimated (12.11 ± 2.73) years when compared to the real age in males with a mean difference (0.04 ± 0.86 year). Also, in males skeletal age was overestimated in all age groups except in group 15 and 17. The MAE was less than two years among the whole male sample (1.02 years).

In females; the age was underestimated by mean difference of (0.15 ± 1.32 year) when compared to chronological age. Also, Greulich and Pyle method underestimated the age in all age groups except group 13, 15, 16, 17. The MAE was less than two years among the whole female sample (1.38 years).

## Discussion

There is an increasing number of cases requiring precise age estimation in living people who do not have a verified date of birth. This is a result of increased migration and territorial mobility in the Middle East^1,2^. In Egypt**,**delinquent children have been of great concern as the criminal law exempts children less than 18 years old from criminal responsibility^15^.

Dental and skeletal maturity can be widely used for age estimation. The term “accuracy” is defined as “the closeness of the difference between the estimated age and chronological age to zero”^1^.

A one-year difference or less was regarded as an “excellent finding” or “accurate” in dental age estimation. Similarly, a two-year discrepancy was labeled as "inaccurate," implying that such differences would be unacceptably large and of little use in forensic age prediction of subadult populations^1^.

Willems et al. simplified the original Demirjian method so the predicted ages could be calculated straight from updated maturity scores. The resulting sum score directly provides the estimated age and results in more accurate estimates^8,9^.

Willems method was applied to a sample of Egyptian children aged from 5 to 16 years old by El-Bakary et al. (2010) et al. Mansoura^6^. The method yielded nearly similar results to the present study, as it reported a mean difference of 0.29 years among males and 0.14 years among females. Yet, it overestimated the calculated age compared to the chronological age. This discrepancy may be due to the different age structures of the studied sample; they studied children with younger age groups and a larger sample size.

A summary of the difference between dental age and chronological age in the different studies in which Willems method was applied to various populations from 2010 to 2020 is illustrated in Appendix I.

In the present study, there was a strong correlation between the dental age estimated by the Cameriere method and real age; however, there was a significant difference between the estimated age and the chronological age in both females and males.

Similar to the present results, the prediction of age by the Cameriere method revealed an average underestimation by a mean difference of 0.49 years for males and 0.26 years for females as reported by El-Bakary et al. when applying this method among Egyptian children in Mansoura in 2010. Yet, they revealed lower mean differences in both sexes^6^.

According to the current study; Willems method is considered more accurate compared to Cameriere method in estimating age in Egyptian children whether males or females. Many studies that used more than one method for dental age estimation concluded the same results^9^.

Various researchers found that when the Willems approach was used on different populations, it produced more accurate and trustworthy findings than any other previously documented radiographic method of estimating dental age. Therefore, the Willems technique has gained popularity as a more accurate predictor of age^3,6,9^.

In the present work, the estimated age utilizing the Greulich and Pyle approach was overestimated by 0.04 years when compared to the chronological age in males. On the other hand, Females’ bone age was underestimated by 0.15 years on average. The MAE for both sexes was within two years.

The discrepancy between the current findings and those reported for other populations is most likely attributable to biological diversity between children of various ethnic backgrounds. The effect of ethnicity on general development including dental and skeletal development is still poorly understood. Some authors contend that ethnic disparities in skeletal growth patterns exist at specific ages, while others attribute differences in the development rates to the individuals’ socioeconomic status or nutrition. Furthermore, sample size, range of age of the sample, age categories of the samples, and the applied statistical approach, could all play a role in the reported differences^15^.

Despite the reported accuracy of these methods; a single method may not be sufficient for precisely determining the age of a subject. To ameliorate diagnostic accuracy, multiple age estimation techniques should be carried out on each individual^16^.

Therefore, physicians are advised to use all available maturity indicators for a more accurate estimate of age according to “The Study Group on Forensic Age Diagnostics”. The use of different techniques could obtain a result as close to the subject’s chronological age as possible^17,18^.

Gelbrich et al. concluded that when methods are combined, the overall picture of the individual’s development is better to be evaluated and therefore the average age among all the methods used becomes closer to the chronological age (18).

## Conclusion and recommendation

Based on the results of this research, it can be concluded that, the Willems method as well as the Greulich and Pyle atlas can be applied with acceptable accuracy for the determination of age in the Egyptian population below 18 years old.

However; for the highest accuracy of age estimation, population-specific standards, rather than a universal standard or methods developed on other populations, need to be employed. Moreover; utilizing multiple approaches at once and averaging their outcomes to estimate age produces a more accurate estimate than utilizing a single one. Combining different approaches makes it easier to assess a person’s overall development.

## Electronic supplementary material

Below is the link to the electronic supplementary material.

Supplementary Material 1

## Data Availability

The authors confirm that the data supporting the findings are available from the corresponding author upon reasonable request. Data analysis is provided within the manuscript.
