# Supplementary material for: Age estimation using dental and hand-wrist radiography among a sample of Egyptian children
Source: Sci Rep. 2025 Jan 7;15:1056. doi: 10.1038/s41598-024-83638-3 (PMC11707342; doi:10.1038/s41598-024-83638-3)
Supplement: Supplementary file 1 — Supplementary Material 1 [file 41598_2024_83638_MOESM1_ESM.docx]

**Appendix I: A table demonstrating the Differences between dental age and chronological age in the different previous studies (from 2010 to 2020) at which Willems method was applied upon different populations**

| **Country** | **Author** | **Year** | **Place** | **Sample** | | | **Age range** | **Mean difference**  **DA-CA** | |
| --- | --- | --- | --- | --- | --- | --- | --- | --- | --- |
|  |  |  |  | **Total** | **Males** | **Females** |  | **Males** | **Females** |
| **Egypt** | El-Bakary et al. ^(1)^ | 2010 | Mansoura | 286 | 134 | 152 | 5-16 | +0.29 | +0.14 |
| **Malaysia** | Nik-Hussein et al. ^(2)^ | 2011 | Malaysia | 991 | 504 | 487 | 5-15 | +0.2 | +0.1 |
| **Korea** | Lee et al. ^(3)^ | 2011 | Seoul | 1483 | 754 | 729 | 3-16 | -0.154 | -0.19 |
| **Italy** | Pinchi et al. ^(4)^ | 2012 | Northern,central and southern Italy | 501 | 244 | 257 | 11-16 | +0.28 | +0.44 |
| **Brazil** | Franco et al. ^(5)^ | 2013 | Curitiba | 462 | 205 | 257 | 5-15.9 | +0.24 | +0.04 |
| **Serbia** | Djukic et al. ^(6)^ | 2013 | Serbia | 686 | 322 | 364 | 4-15 | +0.12 | +0.16 |
| **Bosnia-Herzegovina** | Galic et al. ^(7)^ | 2011 | Sarajevo | 1089 | 498 | 591 | 6-13 | +0.42 | +0.24 |
| **Iran** | Javadinejad et al. ^(8)^ | 2013 | Iran | 537 | 264 | 273 | 3.9-14.5 | +0.43 | +0.31 |
| **France** | Urzel et al. ^(9)^ | 2013 | Bordeaux, Mont-de- Marsan, Floirac | 743 | 357 | 386 | 4-15 | +0.14 | -0.09 |
| **India** | Mohammed et al. ^(10)^ | 2014 | South India | 332 | 166 | 166 | 6-16 | -0.69 | -0.08 |
| **India** | Rajeev et al. ^(11)^ | 2018 | South Kerala | 100 | 50 | 50 | 8-18 | -1.64 | -1.7 |
| **Former Yugoslav Republic of Macedonia** | Ambarkova et al. ^(12)^ | 2014 | Macedonia | 966 | 481 | 485 | 6-13 | +0.52 | +0.33 |
| **Venezuela** | Medina et al. ^(13)^ | 2014 | Caracas | 238 | 117 | 121 | 5-13 | +0.29 | +0.01 |
| **India** | Gupta et al. ^(14)^ | 2015 | Faridabad | 70 | 37 | 33 | 9-16 | +0.49 | +0.45 |
| **India** | Patel et al. ^(15)^ | 2015 | Gandhinagar | 180 | 90 | 90 | 6-16 | +0.15 | +0.017 |
| **India** | Mohammed et al. ^(16)^ | 2015 | Visakhapatnam | 660 | 330 | 330 | 6-16 | -0.7 | -0.11 |
| **Pakistan** | Khoja et al. ^(17)^ | 2015 | Pakistan | 403 | 176 | 227 | 8-16.9 | +0.31 | +0.29 |
| **Turkey** | Akkaya et al. ^(18)^ | 2015 | Ankara | 799 | 387 | 412 | 2.2-15.9 | +0.07 | +0.15 |
| **China** | Zahi et al. ^(19)^ | 2016 | North China | 1004 | 392 | 612 | 11-18 | -0.54 | -1.01 |
| **Turkey** | Altan et al. ^(20)^ | 2016 | Hatay | 756 | 378 | 378 | 5-14.6 | +0.4 | +0.26 |
| **Italy** | Pinchi et al. ^(21)^ | 2016 | Florence | 274 | 139 | 135 | 6-17 | +1.11 | +1.26 |
| **Malaysia** | Kumaresan et al.^(22)^ | 2016 | Malaysia | 426 | 179 | 247 | 5-15 | +0.55 | +0.53 |
| **Thailand** | Duangto et al. ^(23)^ | 2016 | Chiang Mai | 441 | 198 | 243 | 6-15 | -0.37 | -0.39 |
| **India** | Thetay et al. ^(24)^ | 2017 | South India | 660 | 330 | 330 | 6-16.99 | -0.65 | -0.12 |
| **Kosovo** | Kelmendi et al. ^(25)^ | 2018 | Kosovo | 1022 | 498 | 524 | 5-14 | -0.14 | -0.24 |
| **China** | Wang et al. ^(26)^ | 2018 | Eastern China | 1622 | 787 | 835 | 11-18.9 | -0.90 | -0.97 |
| **China** | Yang et al. ^(27)^ | 2019 | Central southern China | 1249 | 646 | 603 | 8-16 | -0.44 | -0.54 |
| **Tunisia** | Nesmi et al. ^(28)^ | 2018 | Tunisia | 500 | 241 | 259 | 5-15 | -0.4 | -0.69 |
| **India** | Hegde et al. ^(29)^ | 2019 | Udaipur | 1200 | 699 | 501 | 5-15 | +0.09 | +0.08 |
| **Taiwan** | Shen et al. ^(30)^ | 2020 | Taiwan | 799 | 404 | 395 | 8-15 | -0.22 | -0.12 |

**Appendix II: A table demonstrating the Differences between dental age and chronological age in the different previous studies (from 2010 to 2020) at which Cameriere method was applied upon different populations**

| **Country** | **Author** | **year** | **Place** | **Sample** | | | **Age range** | **Mean difference**  **DA-CA** | |
| --- | --- | --- | --- | --- | --- | --- | --- | --- | --- |
|  |  |  |  | **Total** | **Males** | **Females** |  | **Males** | **Females** |
| Bosnia-Herzegovina | Galic et al ^(7)^ | 2011 | Sarajevo | 1089 | 498 | 591 | 6-13 | -0.02 | +0.10 |
| Iran | Javadinejad et al. ^(8)^ | 2013 | Iran | 537 | 264 | 273 | 3.9-14.5 | -0.27 | -0.11 |
| Colombia | Rivera et al ^(31)^ | 2017 | Colombia | 524 | 274 | 252 | 6-14 | +0.08 | -0.25 |
| Turkey | Gulsahi et al ^(32)^ | 2015 | Turkey | 573 | 275 | 298 | 8-15 | -0.47 | -0.24 |
| Egypt | El-Bakary et al ^(1)^ | 2010 | Mansoura | 286 | 134 | 152 | 5-16 | -0.49 | -0.26 |
| Mexico | De Luca et al ^(33)^ | 2012 | Mexico | 502 | 248 | 254 | 5-15 | 0.00 | +0.10 |
| China | Guo et al ^(34)^ | 2014 | North China | 785 | 388 | 397 | 5-15 | -0.43 | -0.03 |
| Bosnia and Herzegovina | Latić-Dautović et al ^(35)^ | 2017 | Sarajevo | 560 | 255 | 305 | 8-14 | -0.17 | -0.14 |
| South Africa | Angelakopoulos et al ^(36)^ | 2019 | South Africa | 970 | 479 | 491 | 6-14 | -0.7 | |
| South Africa | Angelakopoulos et al ^(36)^ | 2019 | European | 974 | 481 | 493 | 6-14 | -0.57 | |
| Poland | Różyło-Kalinowska et al ^(37)^ | 2020 | Krakow | 121 | 60 | 61 | 5-13 | +0.17 | +0.18 |

**Appendix III: A table demonstrating the Differences between dental age and chronological age in the different previous studies (from 2010 to 2020) at which Greulich and Pyle was applied upon different populations**

| **country** | **Author** | **Year** | **Place** | **Sample** | | | **Age range** | **Mean difference**  **SA-CA** | | |
| --- | --- | --- | --- | --- | --- | --- | --- | --- | --- | --- |
|  |  |  |  | **Total** | **Males** | **females** |  | **males** | **females** | |
| **Pakistan** | Zafar et al^(38)^ | 2010 | Pakistan | 889 | 535 | 354 | 0-18 | +0.1 | -0.19 | |
| **Portugal** | Santos et al^(39)^ | 2011 | Portugal | 230 | 136 | 94 | 12-20 | +0.12 | +0.02 | |
| **Iran** | Moradi et al^(40)^ | 2012 | Iran | 425 | 303 | 122 | 6-18 | +0.37 | -0.04 | |
| **Israel** | Soudack et al ^(41)^ | 2012 | Israel | 679 | 375 | 304 | 0-18 | +0.16 | -0.04 | |
| **India** | Shilpa et al^(42)^ | 2013 | Bangalore | 250 | 124 | 126 | 6-15 | +0.18 | +0.29 | |
| **Pakistan** | Awais et al^(43)^ | 2014 | Pakistan | 283 | 136 | 147 | 0-18 | -1.3 | +0.06 | |
| **Asian American** | Mansourvar et al^(44)^ | 2014 | Asian American | 48 | 48 | - | 1-8 | +0.87 | - | |
| **Hispanic** | Mansourvar et al^(44)^ | 2014 | Hispanic | 43 | 43 | - | 15-18 | +0.37 |  | |
| **Pakistan** | Mughal et al^(45)^ | 2014 | Pakistan | 220 | 139 | 81 | 4.5-9.5 | -1.3 | +0.55 | |
| **India** | Rai et al ^(46)^ | 2014 | India | 150 | 75 | 75 | 5-15 | -0.77 | -0.33 | |
| **Korea** | Kim et al^(47)^ | 2015 | Korea | 212 | 135 | 77 | 7-12 | -0.48 | -0.02 | |
| **India** | Mohammed et al(^48)^ | 2015 | South India | 660 | 330 | 330 | 9-20 | -0.23 | +0.02 | |
| **Brazil** | de Sousa Dantas et al ^(49)^ | 2015 | North Brazil | 150 | 72 | 78 | 5-18 | 0.00 | +0.6 | |
| **Italy** | Santoro et al^(50^ | 2019 | Italians | 102 | 51 | 51 | 4-19 | 0.00 | | |
| **Italy** | Santoro et al^(50)^ | 2019 | Africans (benin) | 102 | 51 | 51 | 4-19 | -0.4 | | |
| **Saudi Arabia** | Alshamrani et al ^(51)^ | 2020 | Saudi Arabia | 420 | 220 | 329 | 1-18 | -0.36 | | +0.13 |

**References**

1. El-Bakary AA, Hammad SM, Mohammed F. Dental age estimation in Egyptian children, comparison between two methods. J Forensic Leg Med. 2010;17(7):363-7. DOI: 10.1016/j.jflm.2010.05.008.
2. Nik-Hussein NN, Kee KM, Gan P. Validity of Demirjian and Willems methods for dental age estimation for Malaysian children aged 5-15 years old. Forensic Sci Int. 2011;204(1-3):208 e1-6. DOI: 10.1016/j.forsciint.2010.08.020.
3. Lee SS, Kim D, Lee S, Lee UY, Seo JS, Ahn YW, et al. Validity of Demirjian's and modified Demirjian's methods in age estimation for Korean juveniles and adolescents. Forensic Sci Int. 2011;211(1-3):41-6. DOI: 10.1016/j.forsciint.2011.04.011.
4. Pinchi V, Norelli GA, Pradella F, Vitale G, Rugo D, Nieri M. Comparison of the applicability of four odontological methods for age estimation of the 14 years legal threshold in a sample of Italian adolescents. J Forensic Odontostomatol. 2012;30(2):17-25.
5. Franco A, Thevissen P, Fieuws S, Souza PH, Willems G. Applicability of Willems model for dental age estimations in Brazilian children. Forensic Sci Int. 2013;231(1-3):401 e1-4. DOI: 10.1016/j.forsciint.2013.05.030.
6. Djukic K, Zelic K, Milenkovic P, Nedeljkovic N, Djuric M. Dental age assessment validity of radiographic methods on Serbian children population. Forensic Sci Int. 2013;231(1-3):398 e1-5. DOI: 10.1016/j.forsciint.2013.05.036.
7. Galic I, Vodanovic M, Cameriere R, Nakas E, Galic E, Selimovic E, et al. Accuracy of Cameriere, Haavikko, and Willems radiographic methods on age estimation on Bosnian-Herzegovian children age groups 6-13. Int J Legal Med. 2011;125(2):315-21. DOI: 10.1007/s00414-010-0515-8.
8. Javadinejad S, Sekhavati H, Ghafari R. A Comparison of the Accuracy of Four Age Estimation Methods Based on Panoramic Radiography of Developing Teeth. J Dent Res Dent Clin Dent Prospects. 2015;9(2):72-8. DOI: 10.15171/joddd.2015.015.
9. Urzel V, Bruzek J. Dental age assessment in children: a comparison of four methods in a recent French population. J Forensic Sci. 2013;58(5):1341-7. DOI: 10.1111/1556-4029.12221.
10. Mohammed RB, Krishnamraju PV, Prasanth PS, Sanghvi P, Lata Reddy MA, Jyotsna S. Dental age estimation using Willems method: A digital orthopantomographic study. Contemp Clin Dent. 2014;5(3):371-6. DOI: 10.4103/0976-237X.137954.
11. Rajeev R. Dental age estimation in children using Willems method and its correlation with chronological age: a digital orthopantomographic study. Hum Biol. 2018;45:211-27.
12. Ambarkova V, Galic I, Vodanovic M, Biocina-Lukenda D, Brkic H. Dental age estimation using Demirjian and Willems methods: cross sectional study on children from the Former Yugoslav Republic of Macedonia. Forensic Sci Int. 2014;234:187 e1-7. DOI: 10.1016/j.forsciint.2013.10.024.
13. Medina AC, Blanco L. Accuracy of dental age estimation in Venezuelan children: comparison of Demirjian and Willems methods. Acta Odontol Latinoam. 2014;27(1):34-41.
14. Gupta S, Mehendiratta M, Rehani S, Kumra M, Nagpal R, Gupta R. Age estimation in Indian children and adolescents in the NCR region of Haryana: A comparative study. J Forensic Dent Sci. 2015;7(3):253-8. DOI: 10.4103/0975-1475.172453.
15. Patel PS, Chaudhary AR, Dudhia BB, Bhatia PV, Soni NC, Jani YV. Accuracy of two dental and one skeletal age estimation methods in 6-16 year old Gujarati children. J Forensic Dent Sci. 2015 Jan;7(1):18. DOI: 10.4103/0975-1475.150298.
16. Mohammed RB, Sanghvi P, Perumalla KK, Srinivasaraju D, Srinivas J, Kalyan US, et al. Accuracy of four dental age estimation methods in southern Indian children. J Clin Diagn Res. 2015;9(1):HC01-8. DOI: 10.7860/JCDR/2015/10141.5495.
17. Khoja A, Fida M, Shaikh A. Validity of different dental age estimation methods in Pakistani orthodontic patients. Aust J Forensic Sci. 2015;47(3):283-92. DOI: 10.1080/00450618.2014.957347.
18. Akkaya N, Yilanci HO, Goksuluk D. Applicability of Demirjian's four methods and Willems method for age estimation in a sample of Turkish children. Leg Med (Tokyo). 2015;17(5):355-9. DOI: 10.1016/j.legalmed.2015.04.003.
19. Zhai Y, Park H, Han J, Wang H, Ji F, Tao J. Dental age assessment in a northern Chinese population. J Forensic Leg Med. 2016;38:43-9. DOI: 10.1016/j.jflm.2015.11.011.
20. Onat Altan H, Altan A, Bilgic F, Akinci Sozer O, Damlar I. The applicability of Willems' method for age estimation in southern Turkish children: A preliminary study. J Forensic Leg Med. 2016;38:24-7. DOI: 10.1016/j.jflm.2015.11.015.
21. Pinchi V, Norelli GA, Pradella F, Vitale G, Rugo D, Nieri M. Comparison of the applicability of four odontological methods for age estimation of the 14 years legal threshold in a sample of Italian adolescents. J Forensic Odontostomatol. 2012;30(2):17-25.
22. Kumaresan R, Cugati N, Chandrasekaran B, Karthikeyan P. Reliability and validity of five radiographic dental-age estimation methods in a population of Malaysian children. J Investig Clin Dent. 2016;7(1):102-9. DOI: 10.1111/jicd.12116.
23. Duangto P, Janhom A, Prasitwattanaseree S, Mahakkanukrauh P, Iamaroon A. New prediction models for dental age estimation in Thai children and adolescents. Forensic Sci Int. 2016;266:583 e1- e5. DOI: 10.1016/j.forsciint.2016.05.005.
24. Thetay AAR, Triveni V, Mohammed RB, Sujai GN, Sailaja S, Paul MMC. Reliability of Various Modified Demirjian Methods for Age Estimation in South Indians?? A Retrospective Digital Orthopantomographic Study. Ann Med Health Sci Res. 2017;7(2):88-95.
25. Kelmendi J, Vodanovic M, Kocani F, Bimbashi V, Mehmeti B, Galic I. Dental age estimation using four Demirjian's, Chaillet's and Willems' methods in Kosovar children. Leg Med (Tokyo). 2018;33:23-31. DOI: 10.1016/j.legalmed.2018.04.006.
26. Wang J, Bai X, Wang M, Zhou Z, Bian X, Qiu C, et al. Applicability and accuracy of Demirjian and Willems methods in a population of Eastern Chinese subadults. Forensic Sci Int. 2018;292:90-6. DOI: 10.1016/j.forsciint.2018.09.006.
27. Yang Z, Geng K, Liu Y, Sun S, Wen D, Xiao J, et al. Accuracy of the Demirjian and Willems methods of dental age estimation for children from central southern China. Int J Legal Med. 2019;133(2):593-601. DOI: 10.1007/s00414-018-1924-3.
28. Nemsi H, Ben Daya M, Salem NH, Masmoudi F, Bouanene I, Maatouk F, et al. Applicability of Willems methods and Demirjian's four teeth method for dental age estimation: Cross sectional study on Tunisian sub-adults. Forensic Sci Int. 2018;291:281 e1- e9. DOI: 10.1016/j.forsciint.2018.08.007.
29. Hegde S, Patodia A, Dixit U. A comparison of the validity of the Demirjian, Willems, Nolla and Haavikko methods in determination of chronological age of 5-15 year-old Indian children. J Forensic Leg Med. 2017;50:49-57. DOI: 10.1016/j.jflm.2017.07.007.
30. Shen C, Pan J, Yang Z, Mou H, Tao J, Ji F. Applicability of 2 Dental Age Estimation Methods to Taiwanese Population. Am J Forensic Med Pathol. 2020;41(4):269-75. DOI: 10.1097/PAF.0000000000000584.
31. Rivera M, De Luca S, Aguilar L, Velandia Palacio LA, Galic I, Cameriere R. Measurement of open apices in tooth roots in Colombian children as a tool for human identification in asylum and criminal proceedings. J Forensic Leg Med. 2017;48:9-14. DOI: 10.1016/j.jflm.2017.03.005.
32. Gulsahi A, Tirali RE, Cehreli SB, De Luca S, Ferrante L, Cameriere R. The reliability of Cameriere's method in Turkish children: a preliminary report. Forensic Sci Int. 2015;249:319 e1-5. DOI: 10.1016/j.forsciint.2015.01.031.
33. De Luca S, De Giorgio S, Butti AC, Biagi R, Cingolani M, Cameriere R. Age estimation in children by measurement of open apices in tooth roots: Study of a Mexican sample. Forensic Sci Int. 2012;221(1-3):155 e1-7. DOI: 10.1016/j.forsciint.2012.04.026.
34. Guo YC, Yan CX, Lin XW, Zhou H, Li JP, Pan F, et al. Age estimation in northern Chinese children by measurement of open apices in tooth roots. Int J Legal Med. 2015;129(1):179-86. DOI: 10.1007/s00414-014-1035-8.
35. Latic-Dautovic M, Nakas E, Jeleskovic A, Cavric J, Galic I. Cameriere’s European formula for age estimation: A study on the children in Bosnia and Herzegovina. South Eur J Orthod Dentofac Res. 2017;4(2):26-30. DOI: 10.5937/sejodr4-15528.
36. Angelakopoulos N, De Luca S, Palacio LAV, Coccia E, Ferrante L, Pinchi V, et al. Age estimation by measuring open apices in teeth: a new formula for two samples of South African black and white children. Int J Legal Med. 2019;133(5):1529-36. DOI: 10.1007/s00414-019-02096-z.
37. Różyło-Kalinowska I, Kalinowski P, Krasicka E, Galić I, Mehdi F, Cameriere R. The Cameriere method using cone-beam computed tomography (CBCT) scans for dental age estimation in children. Aust J Forensic Sci. 2022;54(3):311-25. DOI: 10.1080/00450618.2020.1789221.
38. Zafar AM, Nadeem N, Husen Y, Ahmad MN. An appraisal of Greulich-Pyle Atlas for skeletal age assessment in Pakistan. J Pak Med Assoc. 2010;60(7):552-5.
39. Santos C, Ferreira M, Alves FC, Cunha E. Comparative study of Greulich and Pyle Atlas and Maturos 4.0 program for age estimation in a Portuguese sample. Forensic Sci Int. 2011;212(1-3):276 e1-7. DOI: 10.1016/j.forsciint.2011.05.032.
40. Moradi M, Sirous M, Morovatti P. The reliability of skeletal age determination in an Iranian sample using Greulich and Pyle method. Forensic Sci Int. 2012;223(1-3):372 e1-4. DOI: 10.1016/j.forsciint.2012.08.030.
41. Soudack M, Ben-Shlush A, Jacobson J, Raviv-Zilka L, Eshed I, Hamiel O. Bone age in the 21st century: is Greulich and Pyle's atlas accurate for Israeli children? Pediatr Radiol. 2012;42(3):343-8. DOI: 10.1007/s00247-011-2302-1.
42. Shilpa PH, Sunil RS, Sapna K, Kumar NC. Estimation and comparison of dental, skeletal and chronologic age in Bangalore south school going children. J Indian Soc Pedod Prev Dent. 2013;31(2):63-8. DOI: 10.4103/0970-4388.115696.
43. Awais M, Nadeem N, Husen Y, Rehman A, Beg M, Khattak YJ. Comparison between Greulich-Pyle and Girdany-Golden methods for estimating skeletal age of children in Pakistan. J Coll Physicians Surg Pak. 2014;24(12):889-93. DOI: 12.2014/JCPSP.889893.
44. Mansourvar M, Ismail MA, Raj RG, Kareem SA, Aik S, Gunalan R, et al. The applicability of Greulich and Pyle atlas to assess skeletal age for four ethnic groups. J Forensic Leg Med. 2014;22:26-9. DOI: 10.1016/j.jflm.2013.11.011.
45. Manzoor Mughal A, Hassan N, Ahmed A. The applicability of the Greulich & Pyle Atlas for bone age assessment in primary school-going children of Karachi, Pakistan. Pak J Med Sci. 2014;30(2):409-11.
46. Rai V, Saha S, Yadav G, Tripathi AM, Grover K. Dental and skeletal maturity- a biological indicator of chronologic age. J Clin Diagn Res. 2014;8(9):ZC60-4. DOI: 10.7860/JCDR/2014/10079.4862.
47. Kim JR, Lee YS, Yu J. Assessment of bone age in prepubertal healthy Korean children: comparison among the Korean standard bone age chart, Greulich-Pyle method, and Tanner-Whitehouse method. Korean J Radiol. 2015;16(1):201-5. DOI: 10.3348/kjr.2015.16.1.201.
48. Mohammed RB, Rao DS, Goud AS, Sailaja S, Thetay AA, Gopalakrishnan M. Is Greulich and Pyle standards of skeletal maturation applicable for age estimation in South Indian Andhra children? J Pharm Bioallied Sci. 2015;7(3):218-25. DOI: 10.4103/0975-7406.160031.
49. de Sousa Dantas I, dos Anjos Pontual A, Almeida MSC, de Lucena MIHM, Beltrão RT, de Moraes Ramos-Perez FM, et al. Evaluation of the Greulich and Pyle method in the determination of bone age and chronological age in a Brazilian population. Derecho y Cambio Social. 2015;12:1-14.
50. Santoro V, Marini C, Fuzio G, Introna F, De Donno A. A comparison of 3 established skeletal age estimation methods in an African group from Benin and an Italian group from Southern Italy. Am J Forensic Med Pathol. 2019;40(2):125-8. DOI: 10.1097/PAF.0000000000000472.
51. Alshamrani K, Hewitt A, Offiah AC. Applicability of two bone age assessment methods to children from Saudi Arabia. Clin Radiol. 2020;75(2):156 e1- e9. DOI: 10.1016/j.crad.2019.08.029.
